# EFNA4 promotes cell proliferation and tumor metastasis in hepatocellular carcinoma through a PIK3R2/GSK3β/β-catenin positive feedback loop

**DOI:** 10.1016/j.omtn.2021.06.002

**Published:** 2021-06-12

**Authors:** Junhao Lin, Chunting Zeng, JiaKang Zhang, Zhenghui Song, Na Qi, Xinhui Liu, Ziyan Zhang, Aimin Li, Fengsheng Chen

**Affiliations:** 1 Cancer Center, Integrated Hospital of Traditional Chinese Medicine, Southern Medical University, Guangzhou 510315, China; 2 Cancer Center, Southern Medical University, Guangzhou 510515, China; 3 Research Center of Carcinogenesis and Targeted Therapy, Xiangya Hospital, Central South University, Changsha 410008, China; 4 Department of Pharmacy, Guilin Medical University, Guilin 541004, China

**Keywords:** EFNA4, hepatocellular carcinoma, EPHA2, PIK3R2, feedback loop, proliferation, migration

## Abstract

Rapid tumor progression, metastasis, and diagnosis in advanced stages of disease are the main reasons for the short survival time and high mortality rate of patients with hepatocellular carcinoma (HCC). Ephrin A4 (EFNA4), the ligand of the EPH family, participates in the development of blood vessels and epithelium by regulating cell migration and rejection. In our study, based on bioinformatics analyses, we found that EFNA4 was highly expressed and led to poor prognosis in patients with HCC. We demonstrated that overexpression of EFNA4 significantly promoted HCC cell proliferation and migration *in vivo* or *in vitro*. In addition, knockdown of EFNA4 inhibited the proliferation and migration of HCC cells. Furthermore, EFNA4 was found to directly interact with EPHA2 and promote its phosphorylation at Ser897, followed by recruitment of phosphoinositide-3-kinase regulatory subunit 2 (PIK3R2) and activation of the glycogen synthase kinase-3beta (GSK3β)/β-catenin signaling pathway. Moreover, overexpression of β-catenin further promoted the expression of PIK3R2, which formed a positive feedback loop. The results revealed that abnormal expression of EFNA4 is the main switch of the PIK3R2/GSK3β/β-catenin loop that influenced the proliferation and migration of HCC cells and suggest that EFNA4 is a potential prognostic marker and a prospective therapeutic target in patients with HCC.

## Graphical abstract

**Figure:**
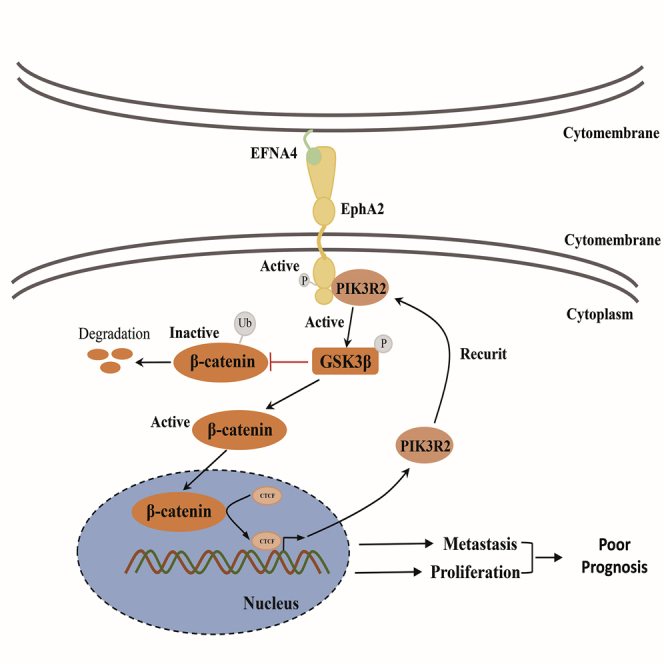

## Teaser

EFNA4 is an oncogene that negatively relates to the clinical prognosis in HCC patients. Abnormal expression of EFNA4 would contribute to HCC proliferation and migration by activating a GSK3β-β-catenin-PIK3R2 positive feedback loop. Thus, clarification of the mechanism of EFNA4 would be helpful for providing a new therapeutic target for HCC patients.

## Introduction

Liver cancer is one of the most common types of malignant tumors, ranking fourth in mortality rate and second in cancer-related mortality of males in 2018 worldwide.^1^ Most patients with liver cancer have entered the advanced stage of disease at the time of diagnosis, and molecular targeting therapy becomes more important for those missing the opportunity for operation. Although tyrosine kinase inhibitors (TKIs) are the first-line treatment for advanced liver cancer patients, TKIs (e.g., sorafenib) are prone to drug resistance. Therefore, the discovery of new treatment options for liver cancer, such as sorafenib enhancers or antibody drug conjugates (ADCs), has become an urgent need for the clinical treatment of liver cancer.

The EPH/ephrin (EPH/EFN) system is widely expressed in various cells by binding to the cell membrane. It plays crucial roles in development, cell proliferation, and differentiation by cell-cell contact, regulation of cell signals and transfer into the nucleus, and stimulation of downstream signaling pathways, which are closely related to the appearance of tumors. Moreover, there are nine types of EPHA receptors and five types of EPHB receptors according to the differences in homology, structural domain, and affinity of gene sequence.^2^ The EPH receptor-interacting proteins (EFN ligands) are divided into eight subtypes, namely five EFNA ligands and three EFNB ligands. The polymer is formed, and bidirectional signaling is active when the receptor binds to the ligands of adjacent cells. EFNA ligands bind to the corresponding EPH receptor, activate the tyrosine kinase in the cytoplasm of the receptor by changing the conformation of EPH, and result in phosphorylation of the corresponding receptor and activation of downstream signaling.^3^ In addition, EFNA ligands activate the relevant surface receptors of their host cells, such as the p75NT receptor (p75NTR).^3^^,^^4^

EFNA4 is mainly expressed in the spleen, lymph nodes, ovary, small intestine, and colon of adults, as well as in the heart, lungs, liver, and kidneys of the fetus. It is involved in the development of neurons, blood vessels, and epithelium by regulating cell migration, rejection, and adhesion. Studies have shown that EFNA4 is involved in the proliferation and metastasis of glioma, ovarian cancer, chronic lymphocytic leukemia, and other tumors.^5^, ^6^, ^7^, ^8^ Moreover, the ADC drug PF-06647263, a conjugate of an EFNA4 monoclonal antibody and calicheamicins, provides a new therapeutic approach for the targeted therapy of patients with advanced breast cancer and ovarian cancer, offering outstanding pharmacokinetics and safety.^9^ However, the role of EFNA4 in the development of hepatocellular carcinoma (HCC) has not been reported yet, and the upstream and downstream regulation of EFNA4 remain unclear. Therefore, the aim of this study was to investigate the role of EFNA4 in the process of HCC occurrence and development.

Based on public database analyses (The Cancer Genome Atlas [TCGA] and Gene Expression Omnibus [GEO]), we found that EFNA4 is highly expressed in HCC and correlated with poorer disease prognosis. Increased expression of EFNA4 promotes the proliferation and migration ability of HCC cells. Mechanistically, overexpression of EFNA4 activates EPHA2 receptor phosphorylation at Ser897. Subsequently, the phosphoinositide-3-kinase regulatory subunit 2 (PIK3R2)/glycogen synthase kinase-3beta (GSK3β)/β-catenin axis influenced the proliferation and migration of HCC cells. Therefore, these findings suggest that EFNA4 could be used as a prognostic marker and that targeting EFNA4 represents a potential therapeutic strategy for patients with advanced HCC.

## Results

### EFNA4 expression is associated with poor prognosis in liver cancer

**Figure 1:**
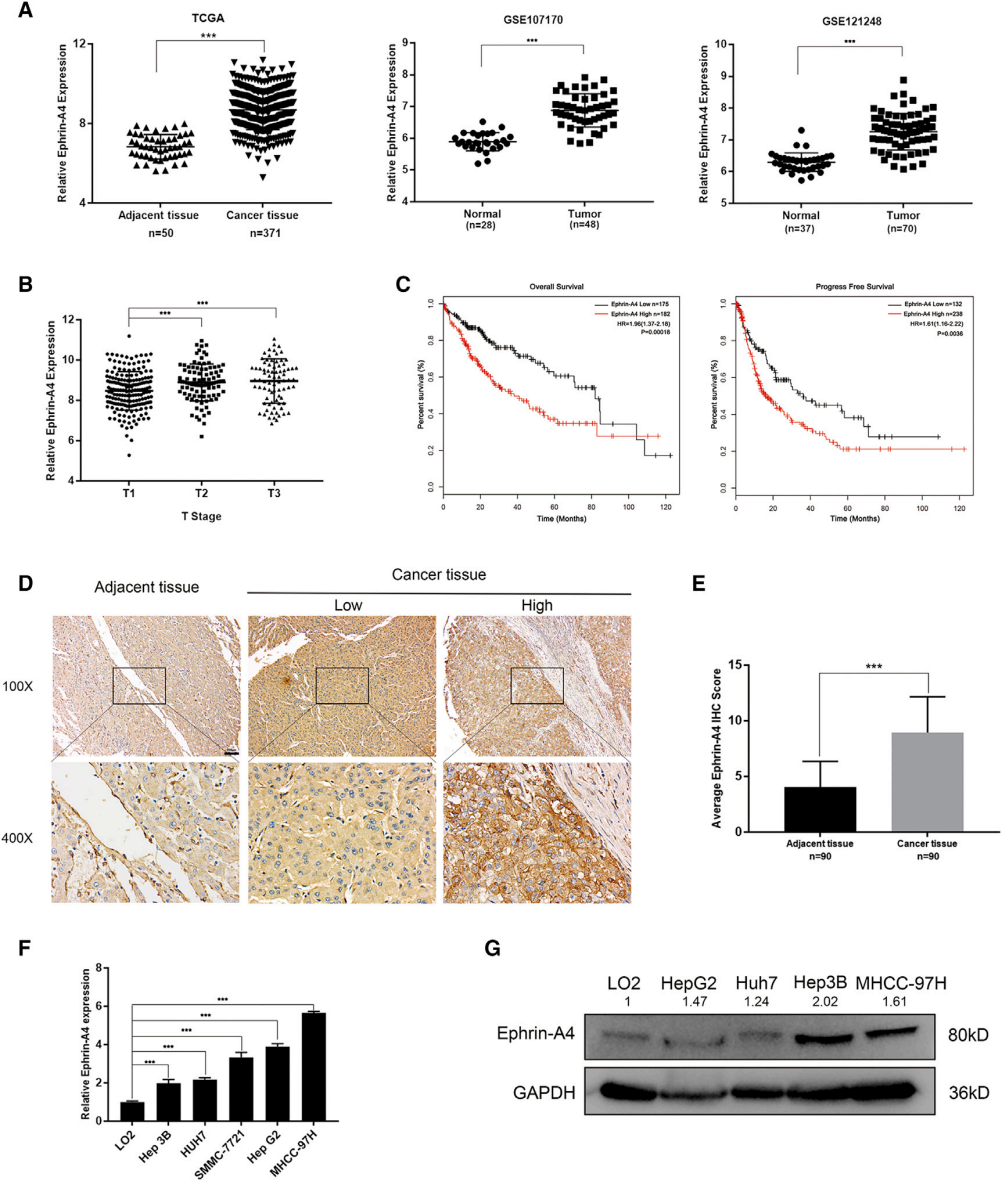
EFNA4 expression is associated with poor prognosis in liver cancer (A) TCGA and two Gene Expression Omnibus datasets (GenBank: GSE121248 and GSE107170) were downloaded for EFNA4 expression analysis. EFNA4 was elevated in hepatocellular carcinoma (HCC) tissues of TCGA dataset (n = 371) compared with adjacent tissues (n = 50). ∗∗∗p < 0.001. (B) Correlation analysis of clinical T staging and EFNA4 expression in TCGA database. (C) Kaplan-Meier analysis of overall survival and progression-free survival among 370 patients with HCC. (D and E) Representative IHC images (D) and average staining scores (E) of EFNA4 expression in 90 pairs of HCC and adjacent tissues. Scale bar, 100 μm. (F and G) EFNA4 expression in HCC cell lines and normal hepatic epithelial cell line at both the RNA and protein levels by quantitative real-time PCR (F) and western blotting (G).

Data from TCGA and GEO databases were extracted and analyzed by bioinformatics methods.^10^ For TCGA database analysis, the results indicated that EFNA4 was significantly overexpressed in patients with liver cancer, and this overexpression was linked to a worse clinical prognosis. Notably, we found that the expression of EFNA4 was positively correlated with TNM staging, and high EFNA4 expression (based on 370 HCC samples) was a significant indicator of poor overall survival (OS) and progress-free survival (PFS; OS: hazard ratio [HR] = 1.96, 95% confidence interval [CI]: 1.37–2.81, p = 0.00018; PFS: HR = 1.61, 95% CI: 1.16–2.22, p = 0.0036) (Figures 1A–1C). Furthermore, gene set enrichment analysis, Kyoto Encyclopedia of Genes and Genomes (KEGG), and Gene Ontology (GO) enrichment were used to analyze data of patients with HCC obtained from the GEO database (GenBank: GSE121248 and GSE107170). The results revealed that EFNA4 expression was negatively correlated with the ability of cells for adhesion, indicating that overexpression of EFNA4 decreases the intercellular adhesion (p < 0.001; enrichment score [ES] = 0.59, 0.46). Moreover, it may affect the occurrence and metastasis of liver tumors by affecting the intercellular connection or participating in the regulation of multiple tumor pathways, such as the phosphatidylinositol 3-kinase (PI3K)-AKT or WNT signaling pathway (Figures S1A–S1C).

To further investigate the correlation between EFNA4 and liver cancer, we analyzed its expression in liver tumor arrays by immunohistochemistry (IHC). As expected, EFNA4 expression in liver tumor tissue was markedly higher than that recorded in adjacent tissue (p < 0.001) (Figures 1D and 1E). Notably, the expression of EFNA4 in 90 tissue samples of liver cancer was related to the expression of alpha-fetoprotein (AFP; χ^2^ test, p = 0.0362) and the risk of vascular invasion (χ^2^ test, p = 0.0319). This finding indicates that liver cancer patients with high EFNA4 expression were more likely to experience tumor metastasis (Table S1). In addition, we compared the basal expression of EFNA4 among normal immortalized liver epithelial cells (LO2) and HCC cells. As shown in Figures 1F and 1G, EFNA4 expression was upregulated in HCC cell lines at both the RNA and protein levels.

### EFNA4 enhances the replication and proliferation of HCC cell lines *in vitro* and *in vivo*

**Figure 2:**
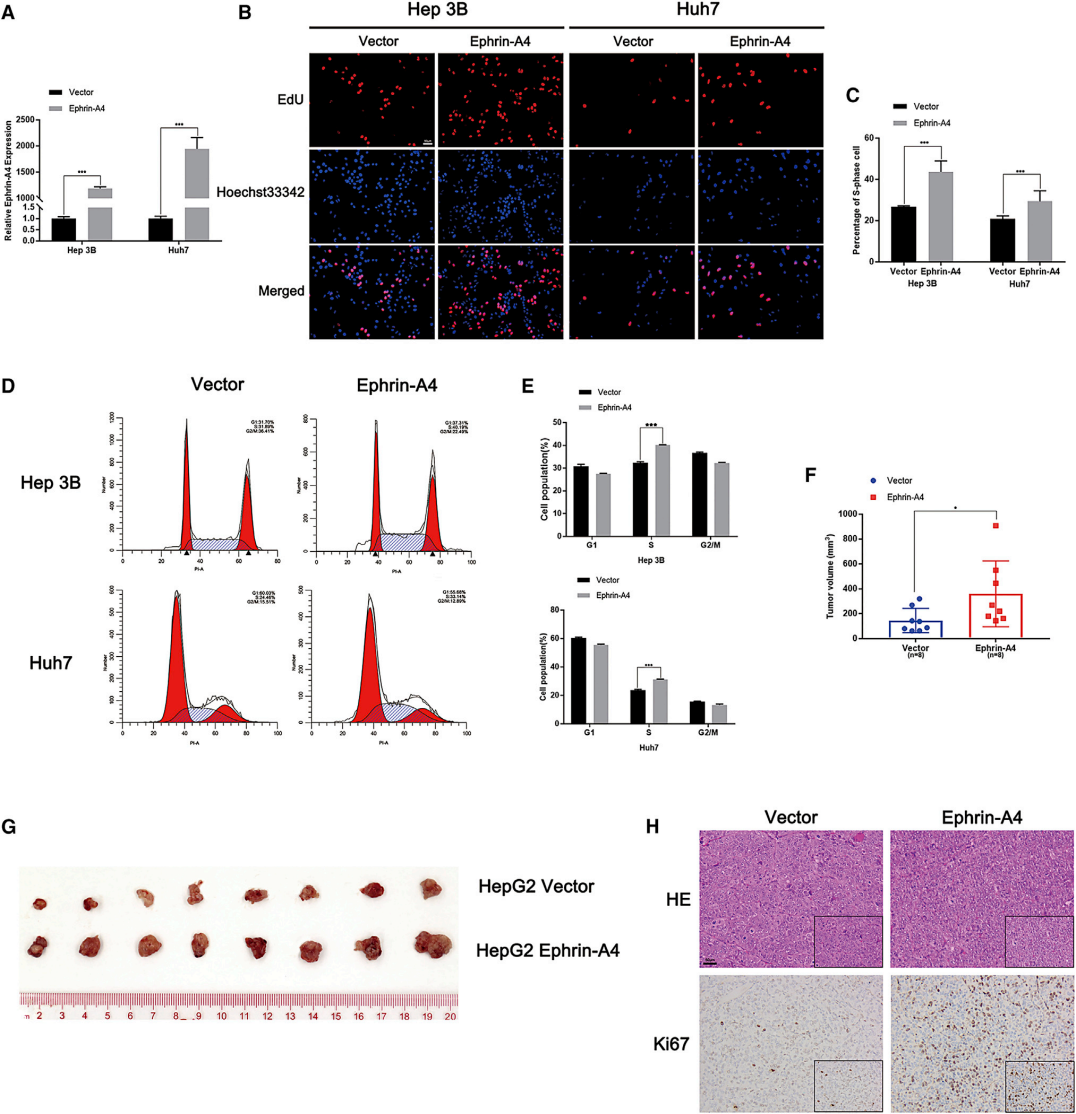
EFNA4 enhances the replication and proliferation of HCC cell lines *in vitro* and *in vivo* (A) Expression of EFNA4 in EFNA4-overexpressing Hep3B and Huh7 cells, as detected by quantitative real-time PCR assays. (B and C) The representative images (B) and quantitative data (C) of the EdU assay in Hep3B and Huh7. Scale bar, 50 μm. (D and E) Representative images (D) and quantitative data (E) from the flow cytometry assays. (F and G) Representative images (G) and quantitative data (F) of the subcutaneous tumor model. ∗p < 0.05, ∗∗p < 0.01, ∗∗∗p < 0.001. (H) Representative images of H&E staining and IHC staining of Ki67. Scale bar, 50 μm.

The present clinical data suggested that EFNA4 may promote tumor progression. To investigate the role of EFNA4 in the pathogenesis and development of liver cancer, we overexpressed EFNA4 in HCC cell lines Hep3B and Huh7. Transfection efficiency was verified by quantitative real-time polymerase chain reaction (PCR) (Figure 2A). 5-ethynyl-2′-deoxyuridine (EdU) assay indicated that, after the overexpression of EFNA4, the number of cells in the DNA replication process of HCC cell lines was significantly increased compared with the vector group (p < 0.001) (Figures 2B and 2C). As shown in Figures 2D and 2E, the percentage of S-phase cells in the empty vector group was 31.89% and 24.46% in Hep3B and Huh7, respectively. Following the overexpression of EFNA4, these values increased to 40.19% and 33.14%, respectively (p < 0.001). Together, the two assays demonstrated that overexpression of EFNA4 could enhance the ability of HCC cells for DNA replication.

We used a mouse subcutaneous tumor model to further confirm the influence of EFNA4 *in vivo*. First, we transfected HepG2 and Huh7 cell lines with EFNA4-overexpressing lentivirus or empty vector lentivirus. After successful transfection, the cells presented green fluorescence, whereas EFNA4 overexpression was confirmed by quantitative real-time PCR (Figures S2A and S2B). Subsequently, the successfully transfected tumor cells were injected into the right groin of the nude mice. Subcutaneous tumor formation assay showed that the mice in the EFNA4 overexpression group showed a larger tumor volume than those in the empty vector group (p < 0.05) (Figures 2F and 2G; Figures S2C−S2E). Further, IHC and hematoxylin and eosin (H&E) staining was performed on the tumor tissue. Compared with the empty vector group, the tumor proliferation index Ki67 was significantly increased in the EFNA4 overexpression group (Figure 2H; Figure S2F). In conclusion, overexpression of EFNA4 increases the ability of HCC cells for DNA replication and proliferation.

### Downregulation of EFNA4 inhibits HCC cell replication and proliferation

To further investigate the biological role of EFNA4, small interfering RNA (siRNA) technology was used to inhibit is expression in HCC cell lines HepG2 and MHCC-97H. The transfection efficiencies were verified by quantitative real-time PCR (Figure S2G). As shown in Figures S2H and S2I, downregulation of EFNA4 expression damaged the DNA replication capacity of HCC cell lines (p < 0.05). As shown by the cell-cycle assay, the decline in EFNA4 expression led to a reduction in the number of S-phase cells (p < 0.05) (Figures S2J and S2K). Therefore, inhibition of EFNA4 expression reduced the ability of HCC cells for DNA replication and proliferation.

### EFNA4 is essential for epithelial-mesenchymal transition (EMT) and migration *in vitro* and *in vivo*

**Figure 3:**
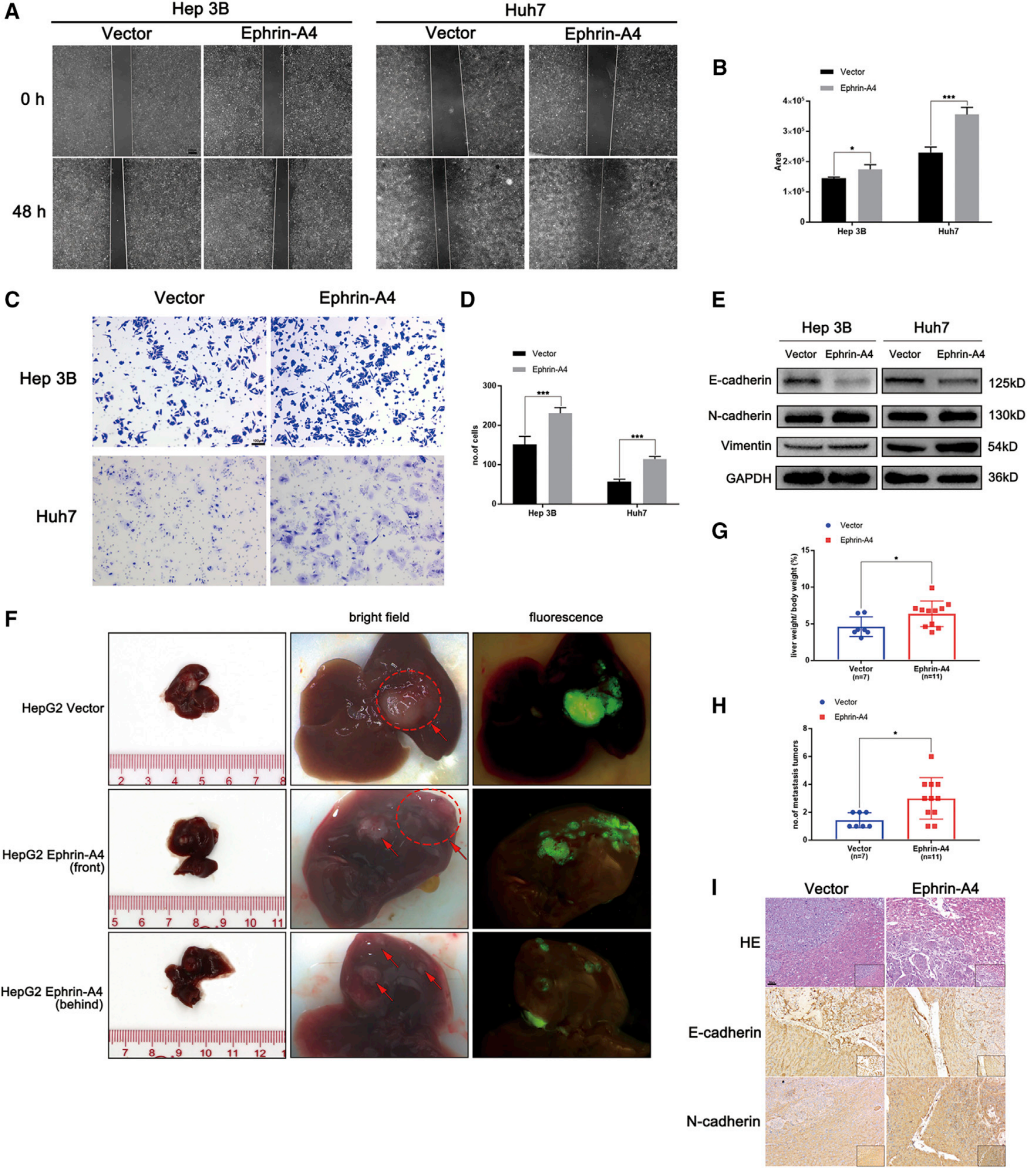
EFNA4 promotes EMT and migration *in vitro* and *in vivo* (A and B) Representative images and quantitative analysis of cell migration based on wound-healing assays. Scale bar, 200 μm. ∗p < 0.05, ∗∗p < 0.01, ∗∗∗p < 0.001. (C and D) Representative images and quantitative analysis of cell migration based on Transwell assays. Scale bar, 100 μm. (E) Analysis of EMT markers by western blotting in EFNA4 overexpression cell lysates. (F and G) Representative images and quantitative analysis of the orthotropic transplantable HCC implantation model; the ellipse represents the site of tumor implantation, and the arrow represents metastasis. (H) The liver weight/body weight ratio analysis between the empty vector group and EFNA4 overexpression group. (I) Representative images of H&E staining and IHC staining of N-cadherin and E-cadherin. Scale bar, 50 μm.

Several research studies found that the abnormal expression of EFNA4 was related to the occurrence of multiple tumor metastases.^11^^,^^12^ We further examined the effect of EFNA4 on the migration ability of HCC cells. For this purpose, Transwell and wound-healing assays were used to investigate the role of EFNA4 overexpression in HCC cells. Functionally, the EFNA4 overexpression group showed a larger healing area compared with the control group (p < 0.05) (Figures 3A and 3B). Moreover, upregulation of EFNA4 promoted the penetration of the basement membrane by tumor cells, thereby facilitating cell migration (p < 0.001) (Figures 3C and 3D). Notably, according to the results of the western blotting analysis, upregulation of EFNA4 assisted HCC cell lines Hep3B and Huh7 in acquiring a mesenchymal phenotype, as mesenchymal markers (N-cadherin and vimentin) were significantly upregulated, and the epithelial marker E-cadherin was significantly downregulated. Overexpression of EFNA4 in HCC cells may induce EMT (Figure 3E).

To verify the effect of EFNA4 overexpression on the ability of tumors for metastasis *in vivo*, HCC cells with EFNA4-overexpressing lentivirus or empty vector lentivirus were injected into the liver of 6-week-old female nude mice. All mice were euthanized 30 days later to verify the effects of EFNA4 on tumor cell migration. As shown in Figure 3F, the dotted ellipse indicates the site of tumor implantation. More tumor nodules with fluorescence appeared in the liver of mice in the EFNA4 overexpression group. Moreover, the liver weight/body weight ratio in the experimental group was significantly higher than that recorded in the control group, suggesting that overexpression of EFNA4 increases the risk of intrahepatic metastasis (Figures 3G and 3H). Subsequently, IHC and H&E staining was performed on the tumor tissue; N-cadherin was upregulated, whereas E-cadherin was downregulated in the EFNA4 overexpression tumor tissues (Figure 3I). Taken together, upregulation of EFNA4 promotes EMT and migration *in vitro* and *in vivo*.

### Downregulation of EFNA4 inhibits HCC cell EMT and migration

In contrast, we found that the migratory ability of cells was significantly decreased after downregulation of EFNA4 expression. As shown in the wound-healing assay, the downregulation of EFNA4 expression reduced the migration area of HCC cells (p < 0.05) (Figures S3A and S3B) and weakened their migratory ability in the basement membrane (p < 0.001) (Figures S3C and S3D). Notably, inhibition of EFNA4 expression increased the expression of E-cadherin in HepG2 and MHCC-97H cell lines, whereas it downregulated the expression of N-cadherin (Figure S3E). In conclusion, downregulation of EFNA4 expression inhibited the EMT and migratory ability of HCC cells.

### EFNA4 promotes phosphorylation of EPHA2 at Ser897 and targets it to activate PIK3R2

**Figure 4:**
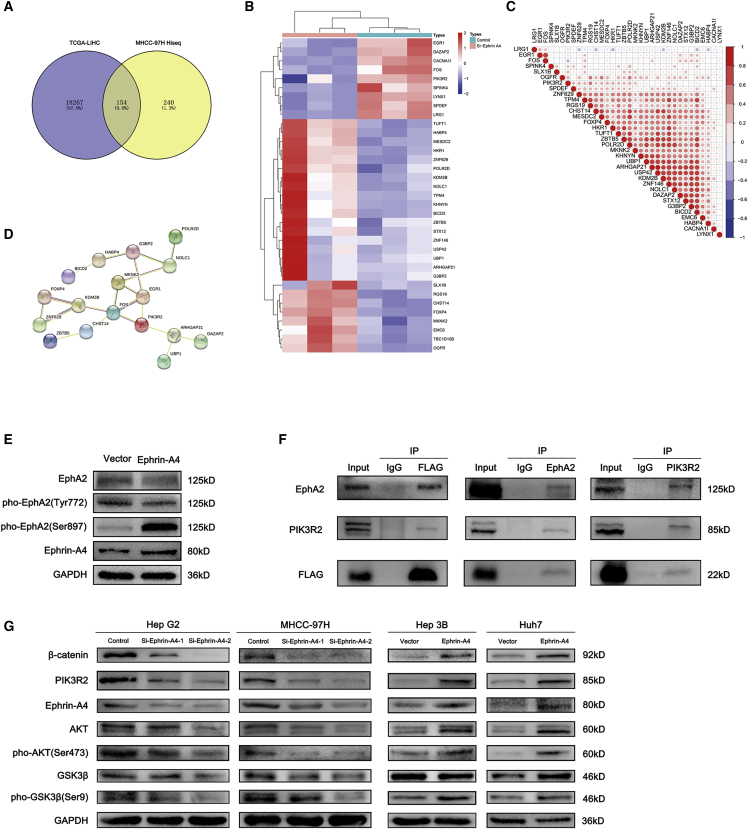
The EFNA4-EPHA2-PIK3R2/GSK3β/β-catenin axis promotes DNA replication and migration of HCC cells (A) Venn diagram of the overlap analysis of EFNA4-related molecules in TCGA database (|R| > 0.2, p < 0.05) and the results of high-throughput sequencing. (B–D) Heatmap (B), correlation analysis (C), and STRING online analysis (D) of the results of the overlap analysis. (E) Analysis of the level of EPHA2 phosphorylation by western blotting using Hep3B cell lysates. (F) EFNA4-EPHA2-PIK3R2 interactions were analyzed by coimmunoprecipitation experiments using Hep3B cell lysates, with an antibody against FLAG-EFNA4, EPHA2, or PIK3R2; interactions were revealed by western blotting. (G) Analysis of the levels of downstream molecules by western blotting using EFNA4-knockdown and EFNA4-overexpressing cell lysates.

We investigated the molecular mechanism through which EFNA4 is associated with HCC proliferation and metastasis. Illumina HiSeq sequence was used to explore the downstream molecules involved in this process and further clarify their specific mechanisms. According to the results of high-throughput sequencing, an overlap analysis of EFNA4-related molecules in TCGA database (|R| > 0.2, p < 0.05) and high-throughput sequencing result was performed. A total of 154 related molecules were found and shown in the Venn diagram (Figure 4A). Following further screening of these molecules (gene counts > 100), a series of methods (e.g., expression calorimetry, correlation analysis, and STRING online analysis) were performed (Figures 4B–4D). As a result, PIK3R2, early growth response 1 (EGR1), and FBJ murine osteosarcoma viral oncogene homologue (FOS) were identified as potential downstream regulatory factors. Subsequently, KEGG enrichment analysis demonstrated that EFNA4 was related to the PI3K-AKT and mitogen-activated protein kinase (MAPK) signaling pathways (Figure S4A). Therefore, PIK3R2 may be the downstream molecule regulated by EFNA4.

To verify our hypothesis, the expression and clinical prognosis of PIK3R2, also known as the PI3K p85β subunit, in patients with liver cancer were analyzed through TCGA database. As shown in Figures S4B−S4D, the expression of EFNA4 was positively correlated with PIK3R2 (R = 0.34, p < 0.05). In addition, PIK3R2 was also significantly upregulated in liver cancer patients (p < 0.001), and the OS time and disease-free survival (DFS) time of patients with high expression of PIK3R2 were also significantly shorter than those with low expression of PIK3R2 (OS: HR = 2, p = 0.0053; DFS: HR = 1.9, p = 0.005). Likewise, interference with the expression of EFNA4 would lead to the decrease of PIK3R2, whereas overexpressed EFNA4 would lead to an opposite result (p < 0.001) (Figure S4E) combined with previous results.^11^^,^^13^ We thus tested and confirmed the interaction among EFNA4, EPHA2, and PIK3R2 by coimmunoprecipitation, immunofluorescence, and western blotting analysis using HCC cell lines Hep3B or Huh7. The experimental results show that there is an interaction among EFNA4, EPHA2, and PIK3R2. Moreover, overexpression of EFNA4 led to phosphorylation of EPHA2 at Ser897 rather than phosphorylating at Tyr772. Follow by EPHA2 activating and PIK3R2 was subsequently recruited to the membrane receptor, which finally stimulating the expression of downstream pathways (Figures 4E and 4F; Figures S4F and S4G). As discussed above, we believe that the EFNA4-EPHA2-PIK3R2 axis may be the mode of action leading to changes in the biological function of HCC cells.

### The EFNA4-EPHA2-PIK3R2 axis regulates the GSK3β/β-catenin signaling pathway

To clarify the effect of the EFNA4-EPHA2-PIK3R2 axis on the downstream signaling pathway, we detected changes in proteins through western blotting analysis. As shown in Figure 4G, downregulation of EFNA4 led to a decrease in PIK3R2, phospho-AKT (Ser473), phospho-GSK3β (Ser9), and β-catenin. In contrast, overexpression of EFNA4 increased the expression of these genes. These results showed that the proliferation and migration of HCC cells may be altered by regulating the EFNA4-EPHA2-PIK3R2 axis associated with the GSK3β/β-catenin signaling pathway.

**Figure 5:**
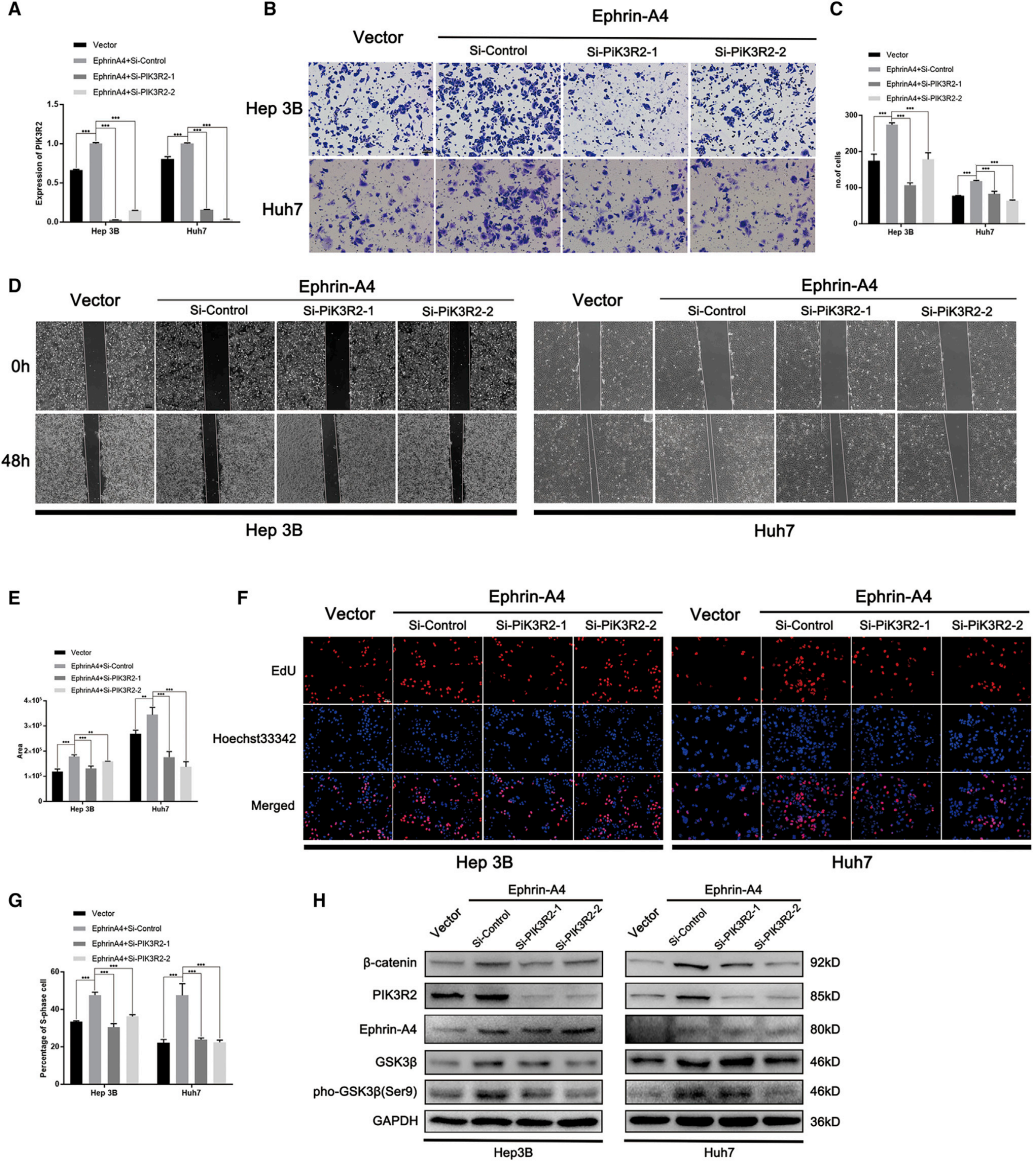
PIK3R2 knockdown reverses the effect of EFNA4 on the proliferation and metastasis of HCC cells (A) PIK3R2 knockdown in EFNA4-overexpressing Hep3B and Huh7 cells, as detected by quantitative real-time PCR assays. ∗p < 0.05, ∗∗p < 0.01, ∗∗∗p < 0.001. (B and C) Representative images and quantitative analysis of cell migration in EFNA4-overexpressing Hep3B and Huh7 cells after knockdown of PIK3R2 based on Transwell assays. Scale bar, 100 μm. (D and E) Representative images and quantitative analysis of cell migration in EFNA4-overexpressing Hep3B and Huh7 cells after knockdown of PIK3R2 based on wound-healing assays. Scale bar, 200 μm. (F and G) Representative images and quantitative data of the EdU assay in EFNA4-overexpressing Hep3B and Huh7 cells after knockdown of PIK3R2. Scale bar, 50 μm. (H) Analysis of the expression of downstream molecules by western blotting using PIK3R2-knockdown and EFNA4-overexpressing cell lysates.

To validate this conclusion, we carried out rescue experiments on key factors in the EFNA4-EPHA2-PIK3R2 axis. Initially, the siRNA technique was used to inhibit the expression of PIK3R2 in HCC cell lines overexpressing EFNA4. Knockdown of PIK3R2 was confirmed by quantitative real-time PCR (Figure 5A). Subsequently, the capacity to migrate, which had been facilitated in HCC cell lines, was restored after knockdown of PIK3R2 (p < 0.001) (Figures 5B–5E). The EdU assay showed that knockdown of PIK3R2 restored the DNA replication ability of HCC cell lines (p < 0.001) (Figures 5F and 5G). The results of western blotting analysis revealed that knockdown of PIK3R2 inhibited the phosphorylation of GSK3 at Ser9, which finally led to the downregulation of β-catenin (Figure 5H).

**Figure 6:**
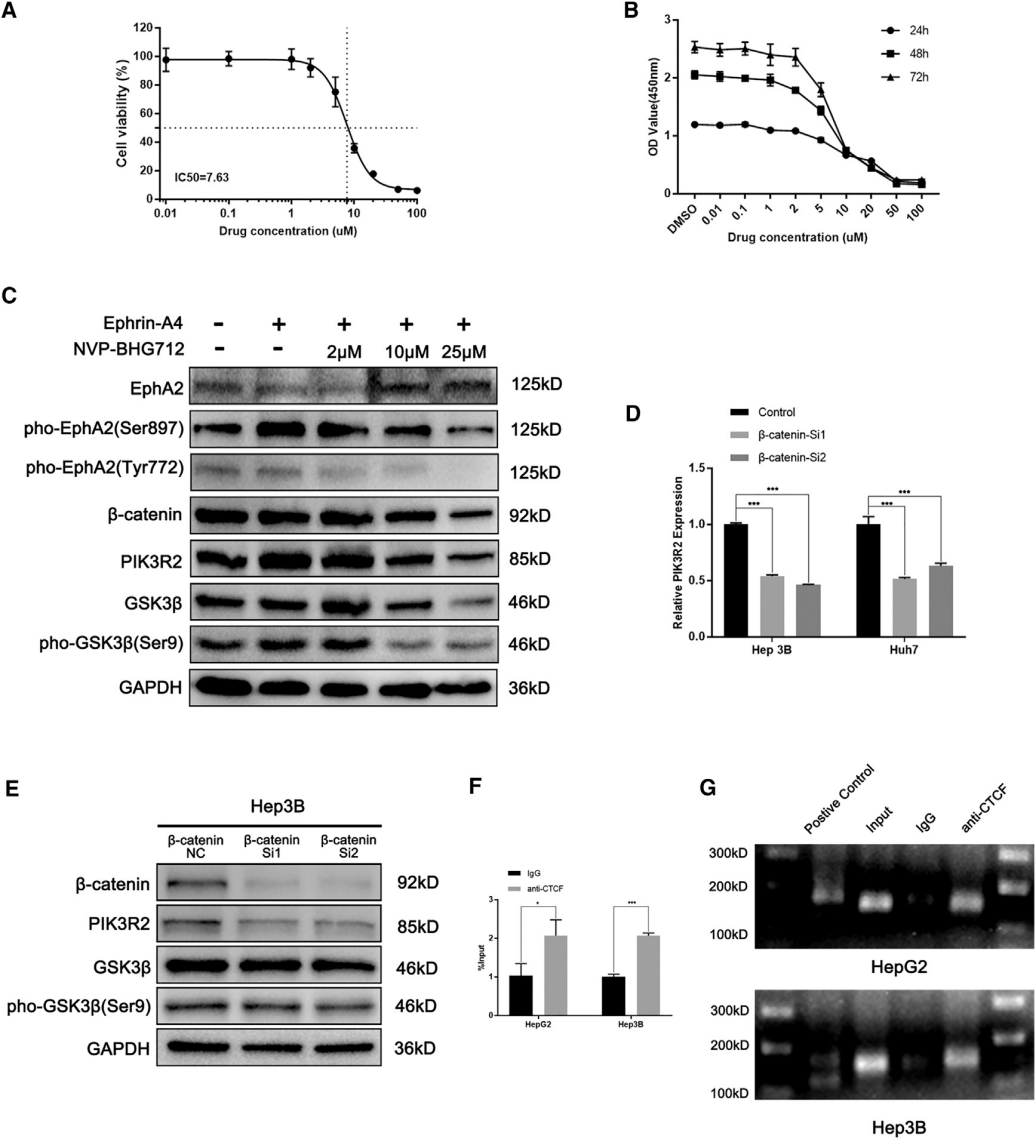
Inhibition of EPHA2 phosphorylation reverses the effect of EFNA4 on downstream molecules of HCC cells, and a feedback loop existed among PIK3R2, GSK3β, and β-catenin (A) The result of the half-maximal inhibitory concentration assay of NVP-BHG712 in Hep3B cells. (B) The result of the CCK-8 assay of NVP-BHG712 in Hep3B cells. (C) Analysis of the expression of downstream molecules by western blotting using different concentrations of NVP-BHG712 in EFNA4-overexpressing Hep3B cell lysates. (D) Expression of PIK3R2 in β-catenin-knockdown HCC cells. ∗p < 0.05, ∗∗p < 0.01, ∗∗∗p < 0.001. (E) Analysis of the levels of downstream molecules by western blotting using β-catenin-knockdown cell lysates in Hep3B. (F and G) Quantitative real-time PCR and PCR gel exhibiting amplification of the CTCF-binding site after the ChIP assay using HepG2 or Hep3B cell lysates with either an antibody against CTCF or IgG.

With the use of the EPH receptor inhibitor NVP-BHG712, we investigated the changes in the EFNA4-EPHA2-PIK3R2 axis and downstream factors after changing the activity of the EPH receptor. NVP-BHG712, an inhibitor of the EPH family tyrosine kinase, inhibits phospho-EPHB4 and phospho-EPHA2.^14^ We investigated the impact of NVP-BHG712 on HCC cells lines in terms of toxicity and proliferation. The results of the half-maximal inhibitory concentration (IC_50_) and Cell Counting Kit 8 (CCK-8) assays revealed that the semi-inhibitory concentration of NVP-BHG712 on HCC cells was 7.63 μM, and with the increase in drug concentration, the cell-survival rate gradually decreased (Figures 6A and 6B). Consistent with previous reports,^14^ phosphorylation of EPHA2 was inhibited by NVP-BHG712 at a concentration >10 μM; thus, we set the dosages as follows: 2, 10, and 25 μM. Proteins were extracted 24 h after treatment, and western blotting analysis was performed to analyze the changes in the expression of downstream molecules. As shown in Figure 6C, following inhibition of the phosphorylation of EPHA2 at Ser897, the expression of downstream molecules PIK3R2, phospho-GSK3β (Ser9), and β-catenin was also inhibited.

### GSK3β/β-catenin and PIK3R2 constitutes a positive feedback loop

Notably, since the abnormal expression of EFNA4, PIK3R2 was recruited to the EPHA2 receptor on the cell membrane for activation. At the same time, the RNA and protein levels of PIK3R2 were also changed. Therefore, we hypothesized that after PIK3R2 activated the GSK3β/β-catenin signaling pathway, β-catenin translocated into the nucleus and promoted the transcription of PIK3R2, which finally formed a positive feedback loop. In order to conform our hypothesis, knockdown of β-catenin was exerted in the HCC cell line (Figure S4H). As expected, after knocking down β-catenin, the expression of PIK3R2 was also inhibited, whereas there were no changes between GSK3β and phospho-GSK3β (Ser9) (Figures 6D and 6E; Figure S4I). Moreover, a chromatin immunoprecipitation (ChIP) assay was designed to explore the influence of β-catenin on the transcription of PIK3R2. Compared with negative control, the existence of the CCCTC-binding factor (CTCF) binding site on the PIK3R2 promoter sequence was confirmed, which suggested that β-catenin may promote the transcription of PIK3R2 by affecting the activation of CTCF (Figures 6F and 6G).

**Figure 7:**
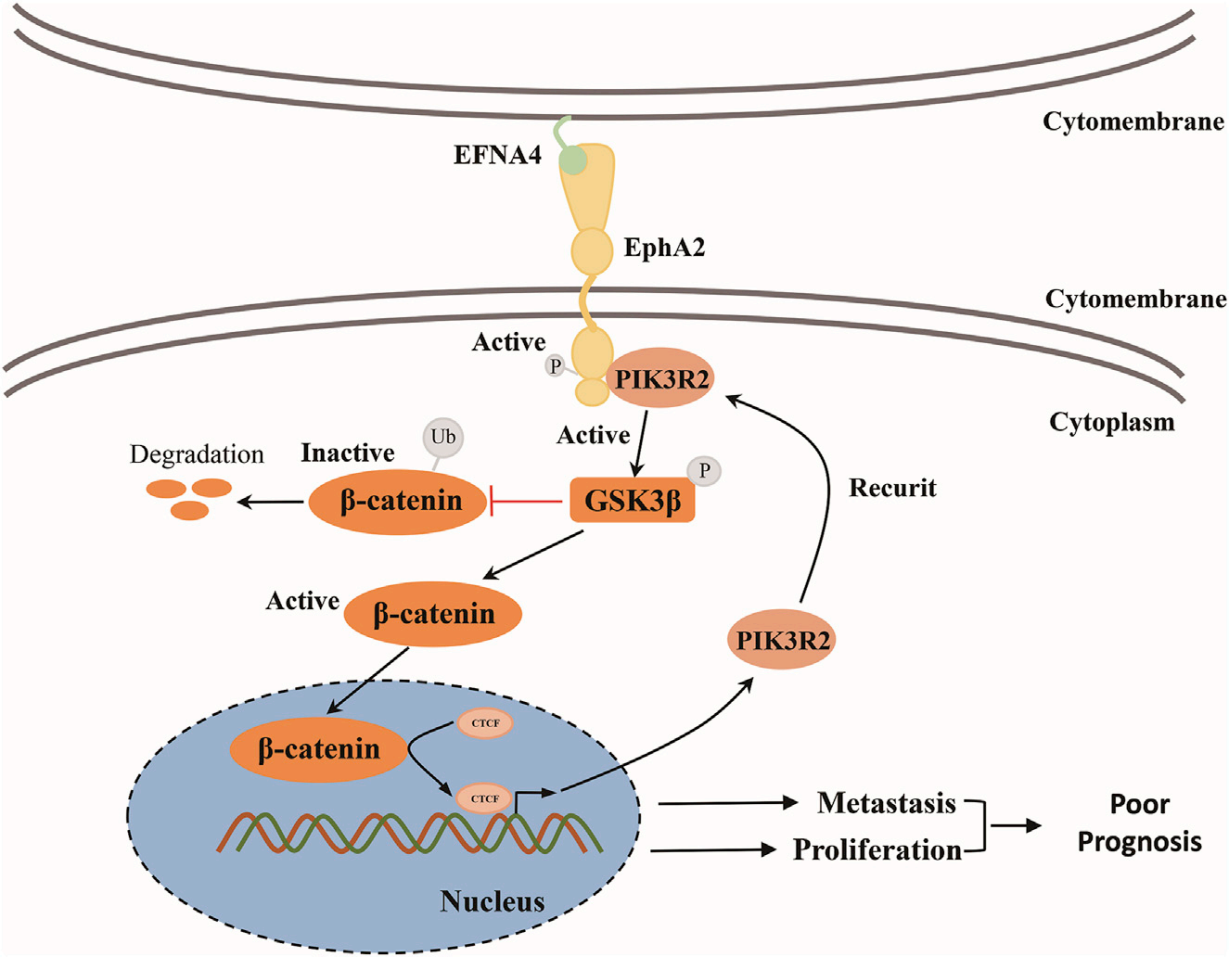
Schematic diagram shows the mechanism between EFNA4 and the PIK3R2/GSK3β/β-catenin positive feedback loop Overexpression of EFNA4 in HCC would active EPHA2 by phosphorylating at Ser897. Moreover, PIK3R2 interacts with EPHA2 and promotes the phosphorylation of GSK3β at Ser9, thus accelerating β-catenin transportation to the nucleus and activating CTCF, which leads to an increase of PIK3R2.

In conclusion, the EFNA4-EPHA2-PIK3R2 axis influences biological functions (e.g., DNA replication and metastasis) of HCC cell lines by regulating the GSK3β/β-catenin signaling pathway. Subsequently, a feedback from β-catenin influenced the transcriptional expression of PIK3R2 (Figure 7), whereas abnormal expression of EFNA4 is the main switch for this process.

## Discussion

Despite a gradual improvement, there is a problem of off-target and drug resistance in first- and second-line targeted drugs for the treatment of liver cancer, which leads to high mortality in patients. Rapid tumor progression and metastasis lead to poor prognosis of patients with tumors. The EPH/EFN system widely participates in the regulation of a variety of biological effects *in vivo*. Several studies found that EFNA4 is involved in the regulation of neuronal, vascular, and epithelial development.^5^, ^6^, ^7^, ^8^ However, in the liver, the high expression of EFNA4 is found only in the early stage of infant development, and, with increasing age, this expression is gradually reduced. The results of our study suggest that the expression of EFNA4 in patients with HCC is a potential prognostic target. Cheng et al.^15^ showed that long-term infection with hepatitis C virus in patients with HCC led to increased expression of EFNA4. According to their bioinformatics analysis, these effects ultimately promoted the proliferation and metastasis of tumor cells.^15^ Thus, we extracted public databases and found that the expression of EFNA4 was significantly upregulated in HCC patients with hepatitis B virus (GenBank: GSE121248) and hepatitis C virus infection (GenBank: GSE107170), followed by activation of some tumor-related signaling pathways.

Several studies suggested that EFNA4 plays a role in the development of tumors. It has been reported that the increased expression of EFNA4 promote the metastasis of human choriocarcinoma cell line JEG-3.^7^ Furthermore, Zhao et al.^16^ found that miR-518a-3p inhibited the metastasis of choriocarcinoma cells by downregulating EFNA4. Aasheim et al.^8^ suggested that dysregulation of EFNA4 induced lymphocytic leukemia by affecting the maturation of B lymphocytes and increasing the number of naive lymphocytes. However, the upstream and downstream factors of EFNA4 are not fully illustrated. In this study, we found that the expression of EFNA4 was significantly increased in tissue sections obtained from patients with liver cancer. Moreover, EFNA4 was positively correlated with the risk of vascular invasion in patients with HCC. Furthermore, *in vitro* and *in vivo* biological function experiments revealed that overexpression of EFNA4 promoted DNA replication, EMT, and tumor migration in HCC cells. Knockdown of EFNA4 expression inhibited the DNA replication and metastasis of HCC cells. These results indicate that the abnormal expression of EFNA4 alters the biological function of liver cells, thereby inducing the occurrence of HCC.

Thus far, the molecular mechanism of EFNA4 remains ambiguous. We thus investigated the mechanism of EFNA4 involved in HCC. Our analysis demonstrated that EFNA4 could bind to the EPHA2 receptor, and overexpression of EFNA4 could activate the phosphorylation of the EPHA2 receptor at Ser897, followed by recruitment of PIK3R2 to the cell membrane. Recent studies suggested that EPHA2 receptors are involved in the regulation of AKT, Yes-associated protein (YAP), and other downstream pathways.^5^^,^^17^ In addition, abnormal activation of the EPHA2 receptor may promote the development of nasopharyngeal cancer,^18^ gastric cancer,^19^ and colon cancer,^20^ whereas PIK3R2 localizes to the cytosol and also concentrates at focal adhesions as well as in the nucleus.^21^^,^^22^ Increasing evidence suggests that upregulation of PIK3R2 triggers cell transformation.^23^^,^^24^ Increased PIK3R2 expression at the cell junction leads to local actin polymerization and the subsequent formation of invadopodia-like structures, which mediate basal membrane degradation and invasion.^21^ We investigated whether the EFNA4-EPHA2 axis promotes HCC cell proliferation and migration by PIK3R2. Our analysis revealed that the expression of PIK3R2 was significantly increased after overexpression of EFNA4. Moreover, as an interaction was shown among EFNA4, EPHA2, and PIK3R2, we further investigated the effects on the downstream signaling pathway. The results showed that the levels of phosphorylated GSK3β and β-catenin were significantly increased after activation of the EFNA4-EPHA2-PIK3R2 axis, whereas inhibition of EFNA4 blocked these effects. After the activation of this axis, phosphorylation of downstream protein GSK3β was increased. As reported, phosphorylation of GSK3β at Ser9 would inhibit the formation of the GSK3β-adenomatous polyposis coli (APC)-AXIN complex, which prevented β-catenin from being degraded by ubiquitin.^25^ Thus, following nuclear translocation of β-catenin, CTCF was activated.^26^ Then CTCF bound to the transcriptional initiating region of PIK3R2, leading to an increase in the transcriptional expression of PIK3R2, which finally formed a positive feedback loop and caused uncontrollable proliferation or metastasis in HCC cells.

PF-06647263 is a conjugate of an EFNA4 monoclonal antibody and the cytotoxic drugs calicheamicins. Compared with using calicheamicins alone, PF-06647263 has shown better efficacy in targeting tumor stem cells and inhibiting tumor growth in breast and ovarian cancer. Moreover, PF-06647263 achieved sustained tumor regression in both triple-negative breast cancer and patient-derived xenograft ovarian cancer *in vivo* through continuous induction of tumor cell regression and reduced initiation of tumor stem cells.^27^ In phase I clinical trial, the PF-06647263 treatment group showed better pharmacokinetic results and safety in patients with metastatic triple-negative breast and ovarian cancer.^9^ Our study provides a theoretical basis for the use of PF-06647263 in patients with liver cancer.

In summary, overexpression of EFNA4 is correlated with poor prognosis in HCC patients. The present evidence indicates that the combination of EFNA4 and EPHA2 wound activate the PIK3R2/GSK3β/β-catenin feedback loop and promotes proliferation and migration in HCC cells, and abnormal expression of EFNA4 is the key point of feedback-loop activation. Therefore, EFNA4 is a potential prognostic marker and a prospective therapeutic target in patients with HCC.

## Materials and methods

### Public data analysis

Bioinformatics data were obtained from TCGA and GEO databases (GenBank: GSE121248 and GSE107170). Significantly differentially expressed genes from HCC and adjacent tissue datasets were screened using the R software (R version [v.]3.5.0). A higher or lower expression of EFNA4, with a p value < 0.05, was regarded as the threshold. The data for the KEGG and GO analyses were obtained from the correlation analysis among EFNA4 and other relative genes in TCGA or the GEO database (|R > 0.3|, p < 0.05).

### Antibodies

Antibodies against EFNA4 (19685-1-AP), E-cadherin (60335-1-Ig), N-cadherin (66219-1-Ig), vimentin (10366-1-AP), EPHA2 (66736-1-Ig), and GSK3β (22104-1-AP) were purchased from Proteintech (Wuhan, China). Antibodies against β-catenin (#8480), AKT (#4691), phospho-AKT (Ser473; #4060), phospho-GSK3β (Ser9; #9323), and EPHA2 (#6997) were obtained from Cell Signaling Technology (Beverly, MA, USA). Antibodies against Ki67 (ab16667), PIK3R2 (ab180967), and CTCF (ab128873) were obtained from Abcam (Cambridge, MA, USA). The antibody against glyceraldehyde 3-phosphate dehydrogenase (GAPDH; AP0063) was purchased from Bioworld Technology (Bloomington, MN, USA). Human EFNA4 antibody (MAB3692) was purchased from Bio-Techne (Minneapolis, MN, USA). Phospho-EPHA2 (Ser897; AP1082) and phospho-EPHA2 (Tyr772; AP0817) were purchased from ABclonal Technology (Wuhan, China). Monoclonal anti-FLAG M2 antibody (1804) was obtained from Merck KGaA (Darmstadt, Germany).

### Cell culture and transfections

The HCC cell lines Hep G2, Hep 3B, Huh7, and MHCC-97H, as well as normal hepatic epithelial cell line (LO2), were obtained from Zhong Qiao Xin Zhou Biotechnology (Shanghai, China). All cells were cultured in cell culture dishes (Guangzhou Jet Bio-Filtration, Guangzhou, China) and maintained in Dulbecco’s modified Eagle’s medium (DMEM), supplemented with 10% (v/v) fetal bovine serum and 5 mg/mL penicillin/streptomycin at 37°C with 5% CO_2_. EFNA4-targeting siRNA and scramble control siRNA were purchased from RiboBio (Guangzhou, China). EFNA4-targeting sequences were as follows: siRNA #1, 5′-GGGCCTCAACGATTACCTA-3′; siRNA #2, 5′-GGAGAGACTTACTACTACA-3′. PIK3R2-targeting sequences were as follows: siRNA #1, 5′-GCACCTATGTGGAGTTCCT-3′; siRNA #2, 5′-GGCCAGACTCAAGAGAAAT-3′. β-catenin-targeting sequences were as follows: siRNA #1, 5′-GCCACAAGATTACAAGAAA-3′; siRNA #2, 5′-GACTACCAGTTGTGGTTAA-3′. The overexpression plasmids pcDNA3.1-EFNA4 as well as the empty vector (pcDNA3.1) were obtained from Sino Biological (Beijing, China). Cell transfection was performed using Lipofectamine 3000 (Thermo Scientific, Waltham, MA, USA) according to the instructions provided by the manufacturer. The expression level of EFNA4 was detected by quantitative real-time PCR.

### Assembly of EFNA4 lentivirus

The EFNA4-overexpressing lentivirus and the empty vector lentivirus were packaged by OBiO Technology (Shanghai, China), and cell transduction was performed according to the instructions provided by the manufacturer. Stable cells were selected using medium containing 2 μg/mL puromycin.

### Total RNA extraction and quantitative real-time PCR

Total RNA was extracted using a cell total RNA isolation kit (Foregene, Chengdu, China) according to the instructions provided by the manufacturer. RNA samples were subsequently reverse transcribed using the PrimeScript RT reagent kit (Takara Biomedical Technology [Beijing], Beijing, China) and amplified by quantitative real-time PCR with a LightCycler480 II system (Roche, Basel, Switzerland) using TB Green Premix Ex Taq II (Takara Biomedical Technology [Beijing], Beijing, China). The expression levels were normalized to those of β-actin, and the relative expression levels were calculated using the 2^−ΔΔCt^ method. The following primer sequences were used: EFNA4: 5′-GAGCTGGGCCTCAACGATT-3′ (forward), 5′-GCTCACAGAATTCGCAGAAGAC-3′ (reverse); PIK3R2: 5′-CTAGCAAGATCCAGGGCGAG-3′ (forward), 5′-ACAACGGAGCAGAAGGTGAG-3′ (reverse); β-catenin: 5′-CTGAGGAGCAGCTTCAGTCC-3′ (forward), 5′-ATTGCACGTGTGGCAAGTTC-3′ (reverse); β-actin: 5′-TGGCACCCAGCACAATGAA-3′(forward), 5′-CTAAGTCATAGTCCGCCTAGAAGCA-3′ (reverse).

### EdU proliferation assay

The EdU proliferation assay was performed with the Cell-Light EdU Apollo 567 *In Vitro* Imaging Kit (RiboBio, Guangzhou, China). Briefly, all cells were inoculated into 96-well plates (1 × 10^4^ cells per well) after transfection with siRNA or plasmid for 24 h, and EdU staining was performed according to the instructions provided by the manufacturer. The number of EdU-positive cells was counted using an inverted fluorescence microscope (Olympus [China], Beijing, China) in three random fields.

### Cell-cycle assay

Cell-cycle assay was performed using the cell-cycle staining kit (MultiSciences [Lianke] Biotech, Hangzhou, China) according to the instructions provided by the manufacturer. The DNA content was analyzed by FACSCalibur flow cytometry (BD Biosciences, Franklin Lakes, NJ, USA), and the percentages of cells within each phase of the cell cycle were determined using the ModFit LT v.4.1.7 software (Verity Software House, Topsham, ME, USA).

### Cell wound-healing and migration assays

The cell wound-healing assay was performed as follows. Cells were seeded and grown into a confluent monolayer in six-well plates. Subsequently, scratches were generated using a pipette tip. After wounding, the cell migration process was visualized using a microscope (Olympus [China], Beijing, China) at 0, 24, and 48 h. Cell migration was assessed through Transwell assays. Briefly, cells in serum-free DMEM were seeded on a membrane (pore size: 8.0 μm) in a 24-well plate (1 × 10^6^ cells per well). DMEM containing 10% fetal bovine serum was added to the lower chamber of each well. After incubation for 24 h, cells in the upper chamber were removed using a cotton swab, and the cells that had reached the underside of the membrane were fixed and stained with crystal violet (0.1% in methylalcohol) for 15 min. The cells located on the underside of the filter (three fields/filter) were counted.

### IHC

Liver cancer tissue arrays (HLivH180Su15) were purchased from Shanghai Outdo Biotech (Shanghai, China). The IHC test kit (PV-9000) for EFNA4 protein expression analysis was purchased from ZsBio (Beijing, China) and utilized according to the instructions provided by the manufacturer. Scores <4 and ≥4 were classified as negative and positive, respectively. Moreover, for cancer tissues, scores ranging from 0 to 6 and >6 were indicative of low and high expression, respectively.

### Illumina Genome Analyzer IIx

The Illumina HiSeq sequence was commissioned by Guangzhou Huayin Medical Laboratory Center (Guangzhou, China). MHCC-97H cells transfected with EFNA4-targeting sequences or the negative control sequences were used for total RNA extraction; each group was analyzed using three individual samples.

### Western blotting

Proteins were resolved by sodium dodecyl sulfate-polyacrylamide gel electrophoresis on 10% or 12.5% precast gels (Epizyme Biotech, Shanghai, China), transferred to polyvinylidene fluoride membranes, blocked with 5% bovine serum albumin for 1 h at room temperature, and incubated with primary antibodies for 12 h at 4°C. Subsequently, membranes were stained with secondary antibodies conjugated with horseradish peroxidase (Bioworld Technology, Bloomington, MN, USA) at 37°C for 1 h. An enhanced chemiluminescence reagent (Millipore, Billerica, MA, USA) was used to visualize the bands, which were detected using the MiniChemi Chemiluminescent Imaging and Analysis System (Sage Creation Science, Beijing, China).

### Coimmunoprecipitation

Coimmunoprecipitation was performed using a Pierce Co-Immunoprecipitation Kit (Thermo Scientific, Waltham, MA, USA), according to the instructions provided by the manufacturer. The final immune complexes were analyzed by western blotting.

### Immunofluorescence colocalization analysis

Cells were inoculated into a 48-well plate (2 × 10^4^ cells per well) after transfection with EFNA4 plasmid for 24 h. The cells were then fixed with 4% paraformaldehyde and incubated with corresponding antibodies and 4′,6-diamidino-2-phenylindole (DAPI). All results were photographed by a confocal laser microscope (Carl Zeiss, Oberkochen, Germany).

### ChIP

The PIK3R2 promoter region sequence was searched in the Ensembl database. JASPAR bioinformatics tools was used for predicting the CTCF binding sites on the PIK3R2 promoter region. ChIP was then performed using a Pierce Agarose ChIP Kit (Thermo Scientific, Waltham, MA, USA), according to the instructions provided by the manufacturer, using anti-CTCF or immunoglobulin G (IgG) antibody. The CTCF bound chromatin was specifically amplified by PCR and analyzed by agarose electrophoresis or quantitative real-time PCR analysis. The following PCR-specific primer sequences were used: CTCF: 5′-TTCAACCCTGGCTTTCTCCG-3′ (forward), 5′-GTTTAGACCCAGAGGCGACC-3′ (reverse).

### IC_50_ and CCK-8 assay

NVP-BHG712 (S2202) was obtained from Selleck Chemicals (Shanghai, China). CCK-8 was purchased from Dojindo Laboratories (Mashikimachi, Japan). Cells were suspended into 96-well plates (3,000 cells per well); when the cells adhered to the plate, inhibitor was added according to the concentration gradient. Spectrophotometric absorbance at 450 nm was measured according to the instructions provided by the manufacturer. Each group was tested at 24, 48, and 72 h.

### Mouse xenograft model

The protocols for the mouse experiments conformed to international regulations for animal care and maintenance and were approved by the Institutional Animal Ethics Committee, Experimental Animal Center of Guilin Medical University.

An orthotropic transplantable HCC implantation model in mice was established to investigate the effect of EFNA4 on intrahepatic metastasis *in vivo*. Moreover, a subcutaneous tumor model was established to explore the effect of EFNA4 on tumor growth. Female nude mice (age: 6 weeks; weight: ∼18 g) were purchased from Hunan SJA Laboratory Animal (Hunan, China). Briefly, 3 × 10^6^ HepG2 or Huh7 cells overexpressing EFNA4 or the empty vector control were used for subcutaneous tumor injection. Furthermore, 2 × 10^6^ HepG2 cells overexpressing EFNA4 or the empty vector control were used for hepatic capsule injection. The subcutaneous tumor model mice were euthanized at 21 days to evaluate the size of the tumors, whereas those of the orthotropic transplantable HCC implantation model were euthanized 30 days later to enumerate the liver metastasis nodules. All tissues were photographed with an inverted fluorescence microscope (Olympus [China], Beijing, China). Following extraction, liver or tumor tissues were fixed in 4% paraformaldehyde. Formalin-fixed, paraffin-embedded sections from each liver tissue sample were stained routinely with H&E and antibodies against Ki67, N-cadherin, or E-cadherin.

### Statistical analysis

Each *in vitro* experiment was performed in at least three independent replicates. The results are presented as the mean ± standard deviation. Student’s t test was used for analysis. OS, PFS, and DFS were determined by Kaplan-Meier survival analysis or Gene Expression Profiling Interactive Analysis.^10^ A χ^2^ test or Fisher’s exact test was utilized to assess the relationship between the expression of EFNA4 and clinicopathological features. All statistical analyses were performed with GraphPad Prism6 (GraphPad Software, San Diego, CA, USA). All statistical tests were two sided, and p < 0.05, p < 0.01, or p < 0.001 denoted statistical significance.

## Supplemental information

Document S1. Figures S1–S4 and Table S1

Document S2. Article plus supplemental information
