# Supplementary material for: EFNA4 promotes cell proliferation and tumor metastasis in hepatocellular carcinoma through a PIK3R2/GSK3β/β-catenin positive feedback loop
Source: Mol Ther Nucleic Acids. 2021 Jun 12;25:328–41. doi: 10.1016/j.omtn.2021.06.002 (PMC8397841; doi:10.1016/j.omtn.2021.06.002)
Supplement: Document S1. Figures S1–S4 and Table S1 [file mmc1.pdf]

OMTN, Volume 25

Supplemental information

EFNA4 promotes cell proliferation and tumor metastasis in hepatocellular carcinoma through a PIK3R2/GSK3β/β-catenin positive feedback loop

Junhao Lin, Chunting Zeng, JiaKang Zhang, Zhenghui Song, Na Qi, Xinhui Liu, Ziyan Zhang, Aimin Li, and Fengsheng Chen

Supplementary Material

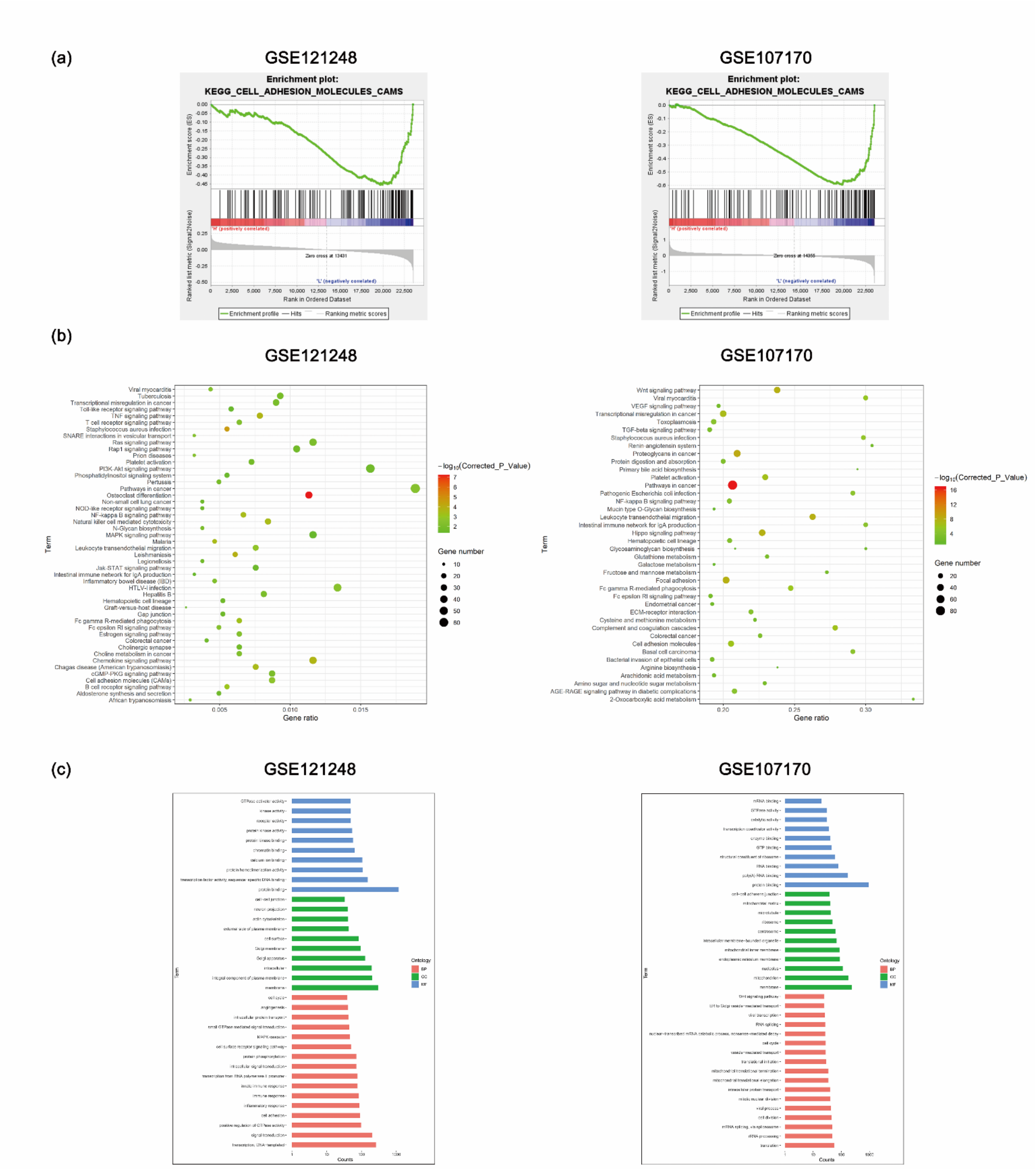

Figure S1 (**A**) GSEA analysis of GEO data sets (GSE121248 and GSE107170). (**B** and **C**) KEGG and GO enrichment of GEO data sets (GSE121248 and GSE107170). Abbreviations: GEO, Gene Expression Omnibus; GSEA, gene set enrichment analysis; KEGG, Kyoto Encyclopedia of Genes and Genomes

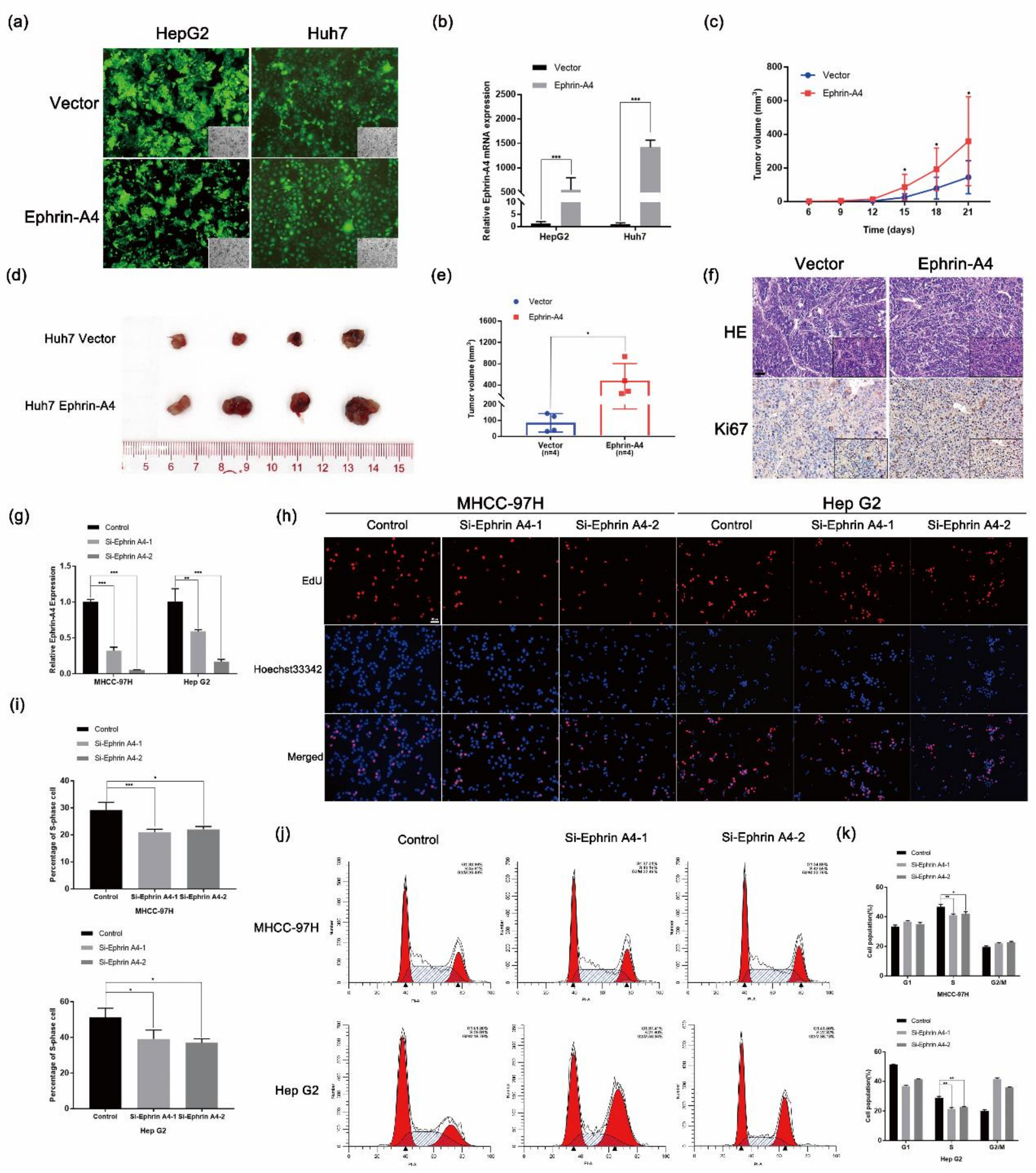

Figure S2 (**A**) Representative images of florescence and bright field of HepG2 and Huh7 cells with EFNA4-overexpressing lentivirus or empty vector lentivirus. (**B**) Expression of EFNA4 in EFNA4-overexpressing HepG2 and Huh7 cells, as detected by quantitative real-time PCR assays. *p < 0.05, **p < 0.01,***p < 0.001. (**C**) Tumor growth curve of the subcutaneous tumor model of HepG2 cells. (**D** and **E**) Representative images and quantitative data of the subcutaneous tumor model using

EFNA4-overexpressing Huh7 cells. (**F**) Representative images of HE staining and IHC staining of KI67, scale bar, 50 μm. (**G**) Expression of EFNA4 in EFNA4-knockdown HepG2 and MHCC-97H cells, as detected by quantitative real-time PCR assays. (**H** and **I**) Representative images and quantitative data of EdU assay in HepG2 and MHCC-97H cells, scale bar, 50 μm. (**J** and **K**) Representative images and quantitative data on flow cytometry assays.

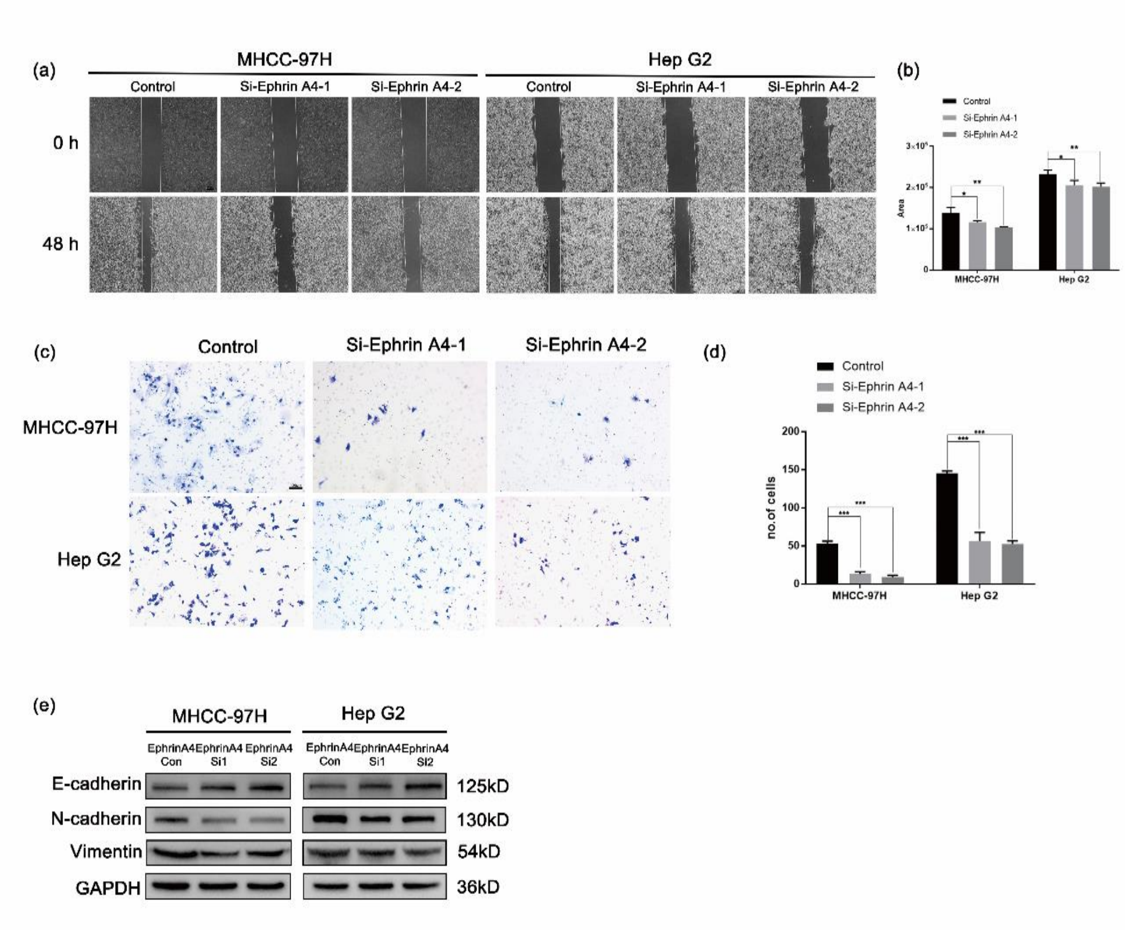

Figure S3 (**A** and **B**) Representative images and quantitative analysis of cell migration based on wound-healing assays using EFNA4-knockdown HepG2 and MHCC-97H cells, scale bar, 200 μm. *p < 0.05, **p < 0.01, ***p < 0.001. (**C** and **D**) Representative images and quantitative analysis of cell migration based on Transwell assays using EFNA4-knockdown HepG2 and MHCC-97H cells, scale bar, 100 μm. (**E**) Analysis of EMT markers by western blotting in EFNA4-knockdown cell lysates.

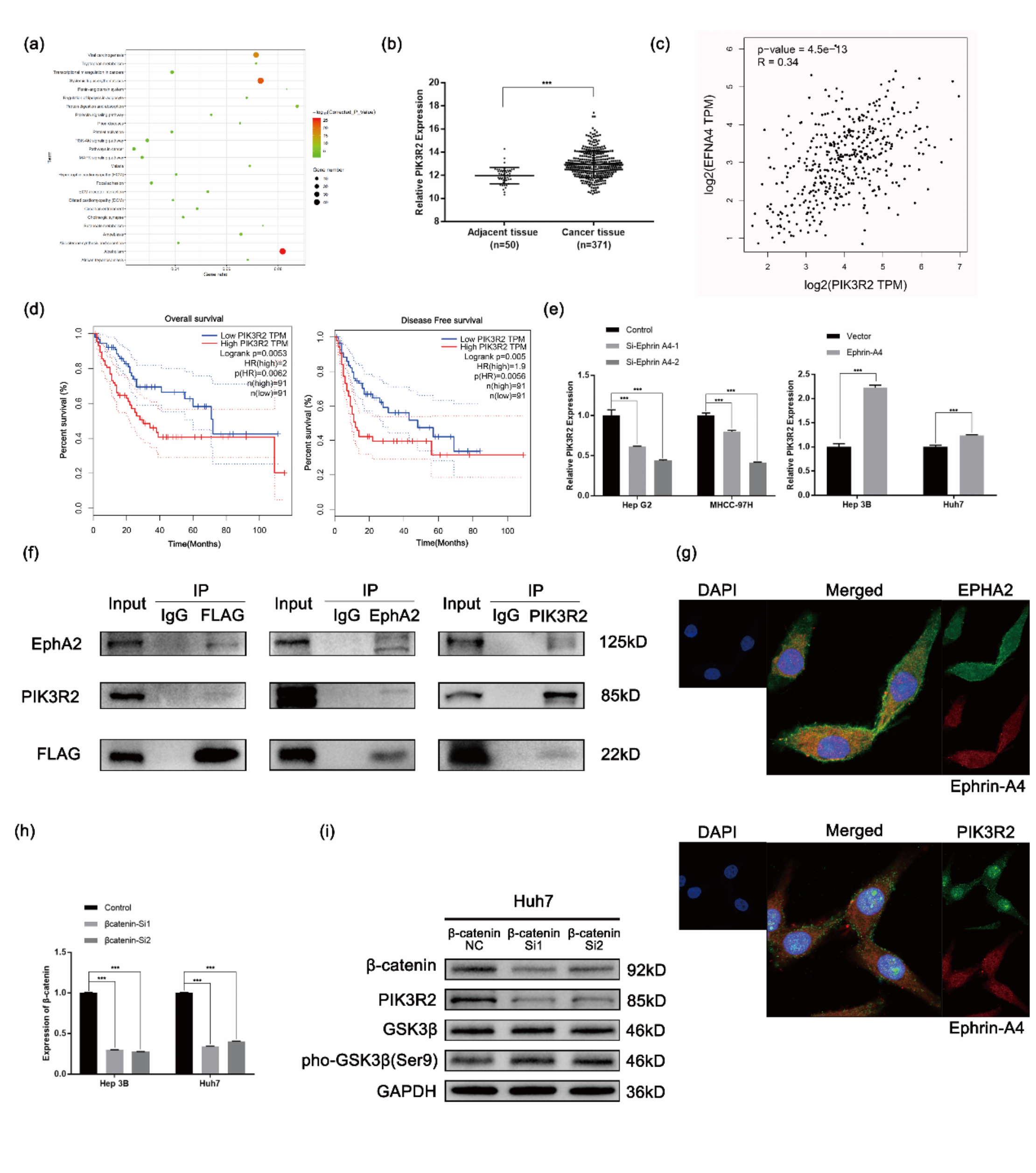

Figure S4 (**A**) KEGG enrichment of 164 altered genes (≥|2-fold change|, P<0.05) after knockdown of EFNA4 in MHCC-97H cells. (**B**) PIK3R2 was increased in HCC tissues of TCGA dataset (n=371) compared with adjacent tissues (n=50). ***p < 0.001. (**C**) Pearson correlation coefficient analysis of EFNA4 and PIK3R2 in TCGA database. (**D**) Analysis of overall survival and disease-free survival among 182 patients with HCC. (**E**) Expression of PIK3R2 in EFNA4-knockdown and EFNA4-overexpressing HCC cells. (**F**) EFNA4-EPHA2-PIK3R2 interactions were analyzed

through Co-IP assay using Huh7 cell lysates, with an antibody against FLAG-EFNA4, EPHA2 or PIK3R2; interactions were revealed by western blotting. (**G**) Immunofluorescence staining of EFNA4 and EPHA2 or PIK3R2 expression and localization in EFNA4-overexpressing Hep3B cell. (**H**) Expression of β-catenin in β-catenin-knockdown HCC cells. (**I**) Analysis of the levels of downstream molecules by western blotting using β-catenin-knockdown cell lysates in Huh7.

Supplementary Table 1 Correlation between EFNA4 expression in tumor tissues and clinicopathological characteristics of patients with liver cancer

Variables	Patients 90	Ephrin-A4 expression(%) Low expression	High expression	P-value
Gender				
Male	80	27(33.8)	53(66.2)	＞0.9999
Female	10	3(30)	7(70)	
Age				
< 60	68	23(33.8)	45(66.2)	＞0.9999
≥ 60	22	7(31.8)	15(68.2)	
HbsAg				
Positive	70	22(31.4)	48(68.6)	0.4194
Negative	19	8(42.1)	11(57.9)	
HCVAb				
Positive	80	30(37.5)	50(62.5)	0.0903
Negative	7	0	7(100)	
TBiL				
⩾17.1	24	6(25)	18(75)	0.3250
＜17.1	65	24(36.9)	41(63.1)	
ALT				
⩾100	7	3(42.9)	4(57.1)	0.6839
＜100	82	27(32.9)	55(67.1)	
AFP				
⩾400	31	6(19.4)	25(80.6)	**0.0362**
＜400	58	24(41.4)	34(58.6)	
Vascular invasion				
Positive	21	3(14.3)	18(85.7)	**0.0319**
Negative	58	24(41.4)	34(58.6)	
Primary tumor (T)				
T_1	58	24(41.4)	34(58.6)	0.0893
T_2	28	5(17.9)	23(82.1)	
T_3	4	1(25)	3(75)	

A χ^2 test or Fisher's exact test was applied to access the associations between the expression of EFNA4 and the clinicopathologic characteristics of liver cancer patients.
